# The association between regular use of aspirin and the prevalence of prostate cancer: Results from the National Health Interview Survey

**DOI:** 10.1097/MD.0000000000003909

**Published:** 2016-06-24

**Authors:** Wan-Ting Huang, Steven R. Erickson, Richard A. Hansen, Chung-Hsuen Wu

**Affiliations:** a School of Pharmacy, College of Pharmacy, Taipei Medical University, Taipei, Taiwan; b Department of Pharmacy, Shuang Ho Hospital, Taipei Medical University, Taipei, Taiwan; c Department of Clinical Pharmacy, College of Pharmacy, University of Michigan, Ann Arbor, MI; d Health Outcomes Research and Policy, Harrison School of Pharmacy, Auburn University, Auburn, AL; e Research Center for Pharmacoeconomics, College of Pharmacy, Taipei Medical University, Taipei, Taiwan.

**Keywords:** Andersen behavioral model of health services use, aspirin, National Health Interview Survey, prostate cancer

## Abstract

Prostate cancer is prevalent with significant morbidity in the United States. Aspirin previously has been found to be associated with reduced carcinogenesis of prostate cells. However, it remains unclear whether regularly taking aspirin could lower the risk of prostate cancer. Therefore, our aim was to examine the association between self-reported regular use of aspirin and the prevalence of prostate cancer in a national sample of the US adult population.

The National Health Interview Survey is an annual survey conducted by the National Center for Health Statistics to investigate health and healthcare use of the US population. The current study is a population-based cross-sectional study using the 2010 National Health Interview Survey data. Adult male respondents who self-reported regularly taking aspirin at least 3 times per week were grouped as regular users. The prostate cancer prevalence was measured by respondents’ self-report of prostate cancer. Multivariable logistic regression models were used to evaluate the association between these 2 factors by adjusting for covariates selected based on Andersen Behavioral Model of Health Services Use.

An estimated 23 million (23.7%) males in the United States reported that they took aspirin regularly. Of them, 5.0% had prostate cancer. Regular aspirin use was significantly associated with a lower self-reported prevalence of prostate cancer after adjusting for predisposing, enabling, and need factors (odds ratio 0.60, 95% confidence interval 0.38–0.94).

Regular aspirin use was found to be significantly associated with a lower self-reported prevalence of prostate cancer in the United States in 2010. Further clinical trials and longitudinal studies are needed to confirm the causality between regular aspirin use and prostate cancer.

## 1 Introduction

Prostate cancer is a global health concern which affects about 1.1 million males in the world.^[1]^ In the United States, prostate cancer accounts for 26% of male cancer patients^[2]^ and is the leading cause of cancer death among males.^[3]^ The estimated medical costs for prostate cancer were $11.9 billion in the United States in 2011,^[4]^ and the projected incidence will reach 228,000 patients in 2030.^[5]^

Previous studies found aspirin use was associated with a reduced risk of several cancers, including colorectal cancer,^[6,7]^ breast cancer,^[8,9]^ and lung cancer.^[10,11]^ However, whether aspirin could have similar benefits in patients with prostate cancer remains unknown. Results from animal and cellular studies indicated that the underlying mechanism of prostate cancer is related to the overexpression of cyclooxygenase-2 (COX-2).^[12–14]^ Aspirin and other nonsteroidal anti-inflammatory drugs (NSAIDs) can inhibit the production of COX-2 and can potentially reduce prostate carcinogenesis.^[15,16]^

Several recent population studies evaluating the association between aspirin use and the risk of prostate cancer found aspirin users had a slightly lower risk of prostate cancer.^[16–18]^ However, these studies did not comprehensively account for risk factors of prostate cancer such as dietary,^[19]^ physical activity, family history,^[20,21]^ or medication use such as the use of finasteride, which could potentially reduce the risk of prostate cancer.^[22,23]^ Therefore, the purpose of this study is to evaluate the association between self-reported regular use of aspirin and the prevalence of prostate cancer in a national sample of the US male adult population. Our hypothesis is that male respondents’ self- reported regular use of aspirin is associated with a lower prevalence of prostate cancer.

## 2 Methods

### 2.1 Data source

We used cross-sectional data from the 2010 National Health Interview Survey (NHIS) to conduct this study. The NHIS is a yearly health survey continuously conducted by the National Center for Health Statistics (NCHS), Centers for Disease Control and Prevention (CDC),^[24]^ and it is widely recognized as the most comprehensive and reliable health survey of the civilian, noninstitutionalized, household population in the United States.^[25]^ Since 1987, every 5 years, the NHIS has added a cancer control supplement to the yearly survey. The supplement consists of 7 sections (diet and nutrition, physical activity, tobacco, cancer screening, genetic testing, family history, and survivorship) of cancer-related health questionnaires which comprehensively obtain health information from respondents who had cancer. The NHIS uses a multistage complex sample design with stratification and clustering to obtain US national estimates.^[24]^ The 2010 NHIS included 89,976 individuals from 35,177 families,^[26,27]^ and the household response rate was 79.5%.^[28]^

### 2.2 Study population and study design

**Figure 1:**
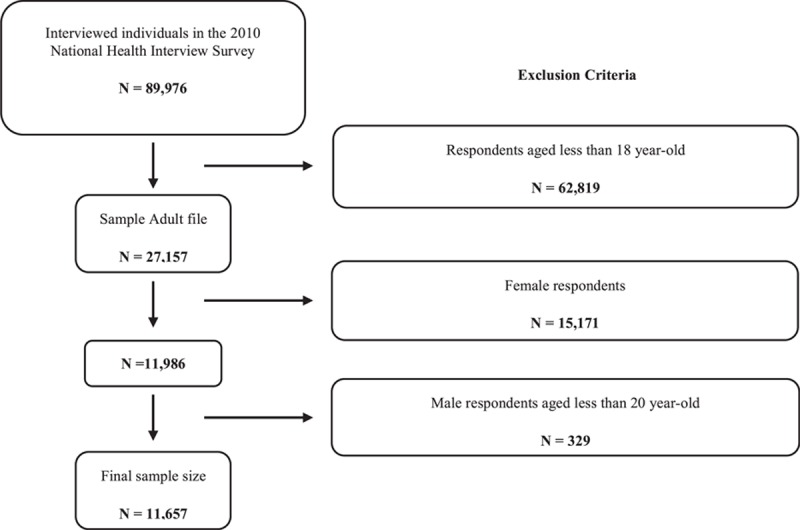
The flow chart of the enrollment process.

This is a population-based cross-sectional study using the Person file, Sample Adult file, and Cancer Control Supplement file of the 2010 NHIS. A total of 11,986 adult male respondents were included. After excluding respondents whose age was less than 20 years, the final sample comprised of 11,657 adult males. Figure 1 shows the details of the enrollment process.

### 2.3 Dependent variables

The dependent variable of this study was self-reported prostate cancer prevalence, which was measured by 2 consecutive questions in the Sample Adult file. The first question asked was, “Have you EVER been told by a doctor or other health professional that you had cancer or a malignancy of any kind?” For respondents who answered yes to this question, a follow-up question was asked: “What kind of cancer was it?” Respondents who reported having “prostate cancer” were defined as patients who self-reported having prostate cancer.

### 2.4 Key independent variable

The key independent variable was whether respondents self-reported regularly taking aspirin, which was measured by a single question administered in the Cancer Control Supplement. The question asked was “Do you now take any of the following medications regularly, that is, at least 3 times a week? Aspirin, Bayer, Bufferin, or Excedrin?” Respondents who answered yes to this question were defined as regular aspirin users. Respondents who answered no to this question were defined as nonregular aspirin users.

### 2.5 Covariates

We used Andersen Behavioral Model of Health Services Use^[29–32]^ to select potential confounders associated with regular aspirin use and the prevalence of prostate cancer. Selected covariates were grouped into predisposing, enabling, and need factors, which we conceptualized to be predictive of prostate cancer.^[29–32]^ A literature search was conducted to guide selection of variables that could influence prostate cancer occurrence and aspirin use. For example, NSAID or COX-2 inhibitor use,^[33,34]^ dietary consumption,^[19,35,36]^ smoking status,^[37–39]^ family history,^[20,40]^ alcohol consumption,^[38,41]^ and exercise^[42,43]^ have previously been reported to be associated with prostate cancer occurrence and were included as covariates in our analysis. These variables were further categorized as predisposing, enabling, and need factors.

Predisposing factors included age (20–50, 51–65, 66–79, and ≥80 years), race/ethnicity (non-Hispanic White, Hispanic, non-Hispanic Black, non-Hispanic Asian, and others),^[39]^ education (less than high school, high school, some college, higher than college), US citizen (yes/no), and cancer-related health beliefs (measured by the self-perceived risk of cancer compared with average men, coded as less likely, about as likely, and more likely).

Enabling factors included insurance (yes/no),^[44]^ family income (measured by the ratio to the poverty threshold: <1.0, 1.00–1.99, and ≥2.0), region of residence (Northeast, Midwest, South, and West), regular finasteride use defined as taking at least 3 times per week (yes/no),^[22,23]^ regular use of nonaspirin NSAIDs or COX-2 inhibitors defined as taking at least 3 times per week (yes/no),^[33,34]^ and antidiabetic drug use (yes/no).^[45–47]^

Need factors included family history of prostate cancer (measured by 2 variables: father had prostate cancer [yes/no] and brother had prostate cancer [yes/no]),^[20,40,48]^ smoking status (current, former, and never),^[37–39]^ alcohol drinking status (current, former, and never),^[38,41]^ frequency of vigorous physical activity (never/unable, less than once per week, 2 times per week, and over 3 times per week),^[42,43]^ nutritional status (measured by the frequency of dietary consumption of red meat,^[36]^ cheese,^[49]^ milk,^[50,51]^ calcium, and vitamin D),^[52,53]^ health status (excellent, very good, good, fair, and poor), numbers of prostate-specific antigen (PSA) tests performed during the past 5 years (never, <5 times, 5–9 times, and ≥10 times),^[54,55]^ body mass index (BMI) (measured as a continuous variable),^[56,57]^ and self-reported diabetes mellitus (yes/no).^[58–60]^

### 2.6 Statistical analysis

For descriptive statistics, we used Student *t* test and Wald chi-square test to describe and compare continuous and categorical patient characteristics between regular aspirin users and nonregular aspirin users. For inferential statistics, simple logistic regression models were used to test the association between each covariate and the prevalence of prostate cancer. A multivariable logistic regression model was used to evaluate the association between regular aspirin use and the prevalence of prostate cancer adjusting for predisposing factors, enabling factors, and need factors. To enhance the robustness of the regression model, we further tested the interaction term between age and regular aspirin use to ensure the interaction term was not significant.

All estimates were weighted to be nationally representative and account for the multistage, complex sample design in the NHIS. The sampling strategy of NHIS is multistage with stratification to form several primary sample units (PSU). After obtaining data from respondents in each PSU, the information was weighted back to obtain the national estimates of the US population. The sample weights were calibrated to 2000 census-based totals for sex, age, and race/ethnicity of the US civilian noninstitutionalized population.^[24,28]^ All data management and analyses were performed using SAS v.9.4.^[61]^ We used the SAS survey procedures (surveymeans, surveyfreq, and surveylogistic) and standard Taylor Series Linearization methods to compute standard errors (SEs) and 95% confidence intervals (CIs). Two-tailed tests with a 0.05 level of significance were used to determine statistical significance. The study was approved as exempt human subjects research by the Taipei Medical University Joint Institutional Review Board, which is an ethics review panel.

### 2.7 Sensitivity analysis

Based on Andersen Behavioral Model of Health Services Use, the predisposing, enabling, and need factors were assumed to be independently associated with prostate cancer.^[29–32]^ Following this assumption, we performed sensitivity analyses by entering covariates (first, predisposing factors; second, enabling factors; and finally, need factors) into the multivariable logistic regression model in a hierarchical pattern to evaluate the relative contribution of each variable.

## 3 Results

Table 1  shows the characteristics of the male population. The estimated US male adult population was 106.6 million in 2010. An estimated 2.5 million male respondents (2.3%) reported that they ever had prostate cancer. The estimated number of regular aspirin users was about 23.4 million (23.7%). Roughly, 60.3% of the male respondents were aged 20 to 49 years and 69.1% were non-Hispanic white. The largest proportion of people lived in the Southern region (35.4%). About 1.3% of male respondents regularly took finasteride, and 14.8% regularly took nonaspirin NSAIDs or COX-2 inhibitors. Of the total sample, 21.7% were current smokers. More than half (52.5%) of the male respondents were either never or were unable to exercise, and 34.4% engaged in regular exercise more than 3 times a week. Approximately 90% of the male respondents never took calcium and vitamin D supplements. Most male respondents (87%) reported being in more than good health status. More than 70% of the male respondents did not receive a PSA test in the previous 5 years. Regarding cancer health beliefs, an estimated 10.7 million US males (11.4%) considered themselves as more likely to get cancer.

**Table 1:**
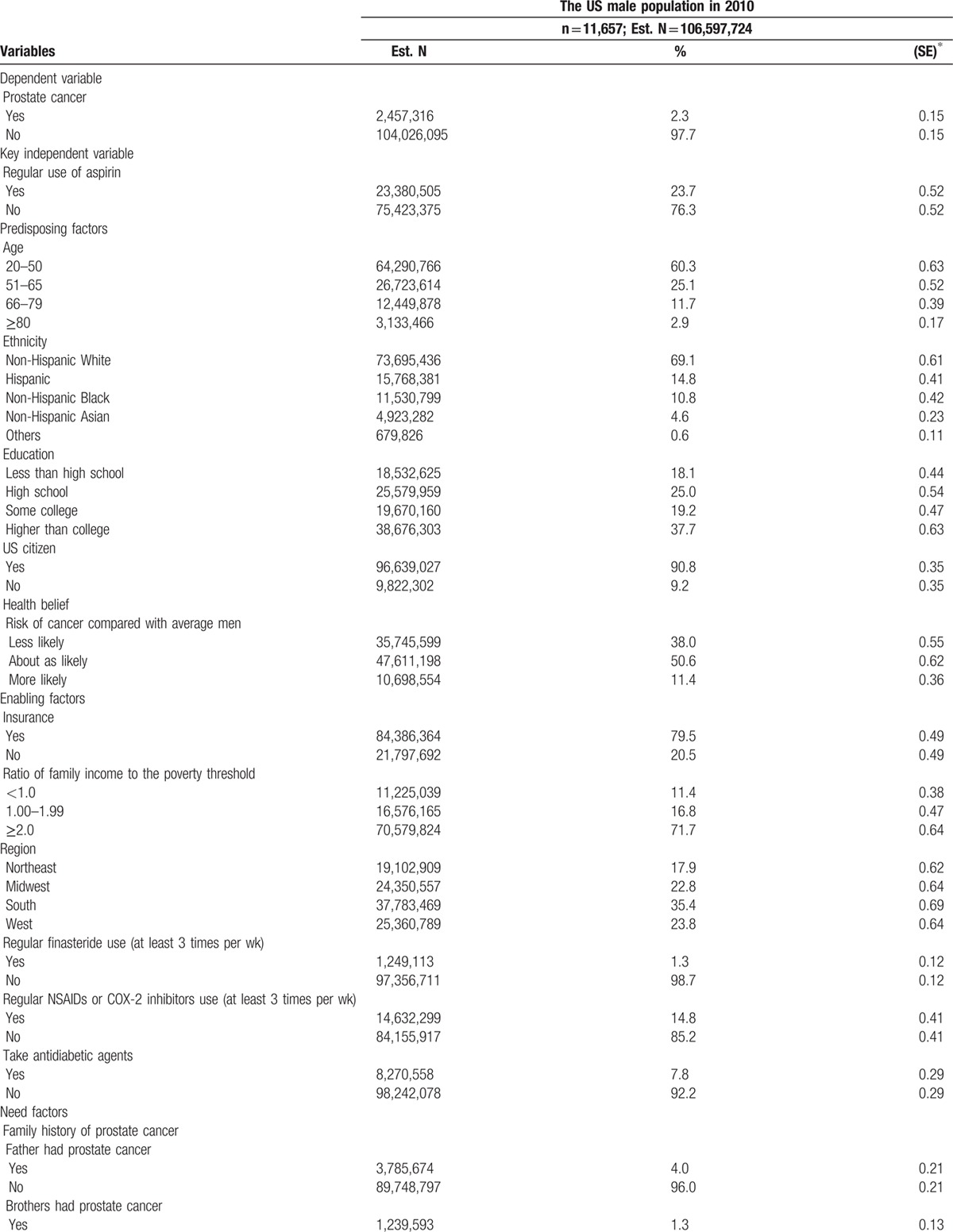
Characteristics of the US male population in 2010.

**Table 1 (Continued):**
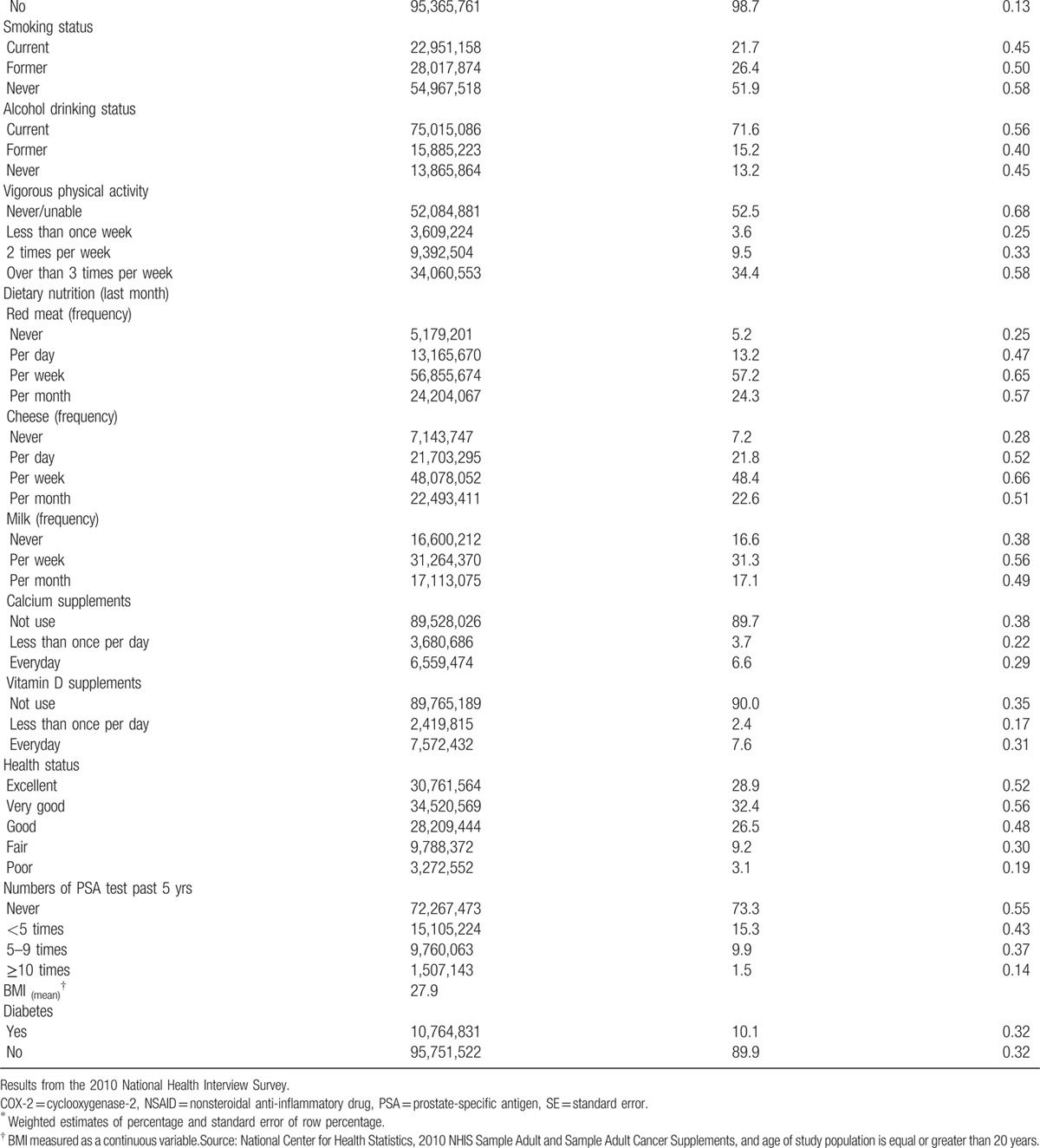
Characteristics of the US male population in 2010.

Table 2  shows the characteristics and comparison of the US male population with and without prostate cancer. Older people were more likely to have prostate cancer. Male respondents who believed that they had a higher risk of getting cancer were more likely to have prostate cancer (less likely: 1.7% vs about as likely: 1.9% vs more likely: 5.8%; *P* < 0.01). Respondents whose brothers had prostate cancer were more likely to report having prostate cancer (20.3% vs 2.0%; *P* < 0.01). Male respondents who were unable or never engaged in vigorous physical activity were significantly more likely to have prostate cancer compared with male respondents with regular physical activity (never/unable: 3.0% vs less than once week: 1.3%, vs 2 times per week: 1.2%, vs over than 3 times per week: 1.9%; *P* < 0.01). Male respondents who took calcium or vitamin D supplements every day were significantly more likely to have prostate cancer. Patients who self-reported better health status were significantly less likely to have prostate cancer. Male respondents who received a PSA test more often were more likely to have prostate cancer, especially those who received the test more than 10 times in the past 5 years.

**Table 2:**
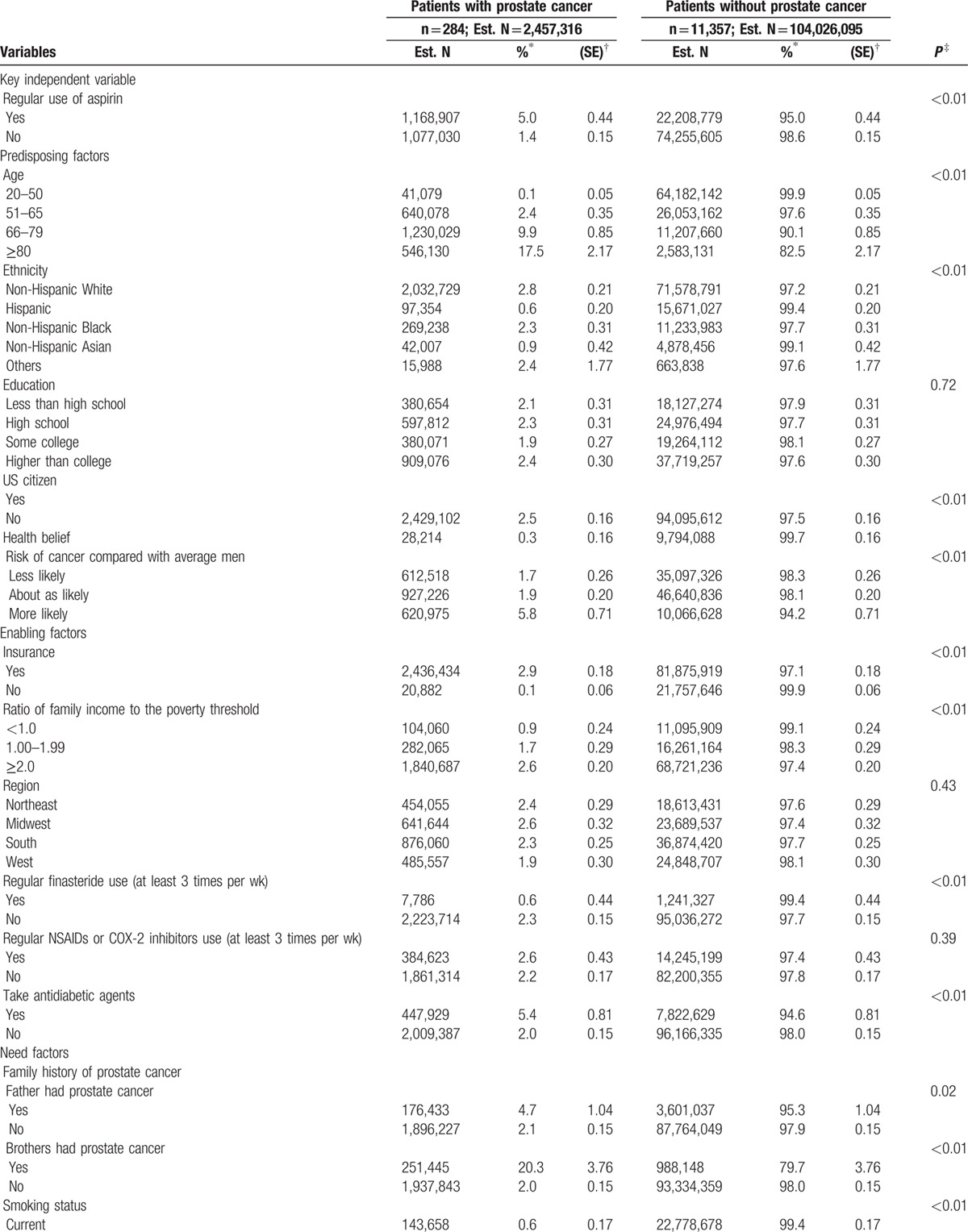
Characteristics of prostate cancer among the US male population in 2010, extrapolated from the sample adult cancer supplement to the National Health Interview Survey.

**Table 2 (Continued):**
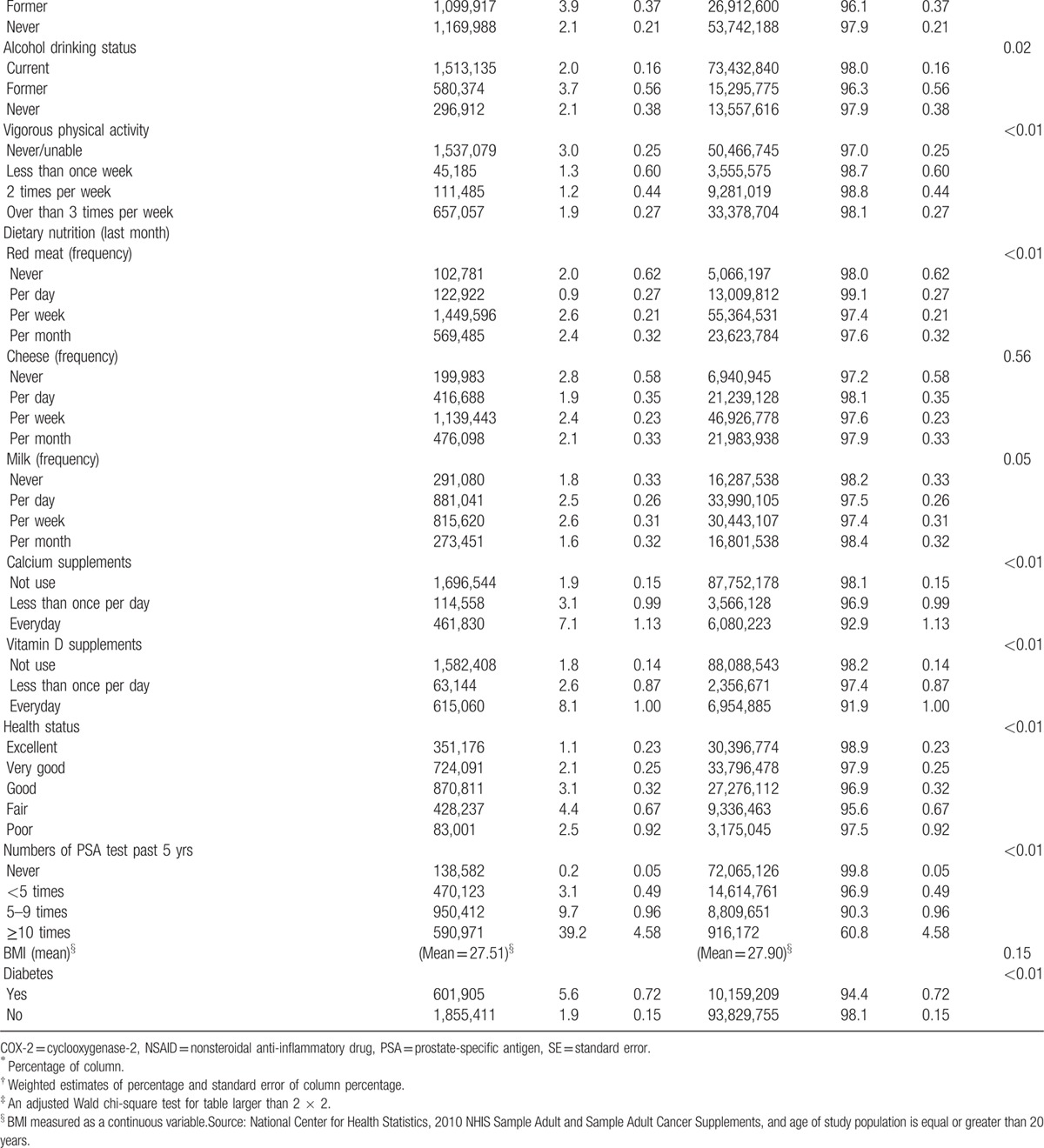
Characteristics of prostate cancer among the US male population in 2010, extrapolated from the sample adult cancer supplement to the National Health Interview Survey.

Table 3  shows the results of the main and sensitivity analyses. Results in the first sensitivity analysis (model 1) showed that regular aspirin use was associated with a lower self-reported prevalence of prostate cancer when compared with nonregular aspirin use after adjusting for predisposing factors, but the result was not statistically significant (odds ratio [OR] 0.95, 95% confidence interval [CI] 0.69–1.31). Results in the second sensitivity analysis (model 2) showed that regular aspirin use was associated with a lower self-reported prevalence of prostate cancer when compared with nonregular aspirin use after adjusting for predisposing and enabling factors, but the result again was not statistically significant (OR 0.86, 95% CI 0.61–1.21). Results in the main analysis (model 3) showed that regular aspirin use was significantly associated with a lower self-reported prevalence of prostate cancer when compared with nonregular aspirin use after adjusting for predisposing, enabling, and need factors (OR 0.60, 95% CI 0.38–0.94).

**Table 3:**
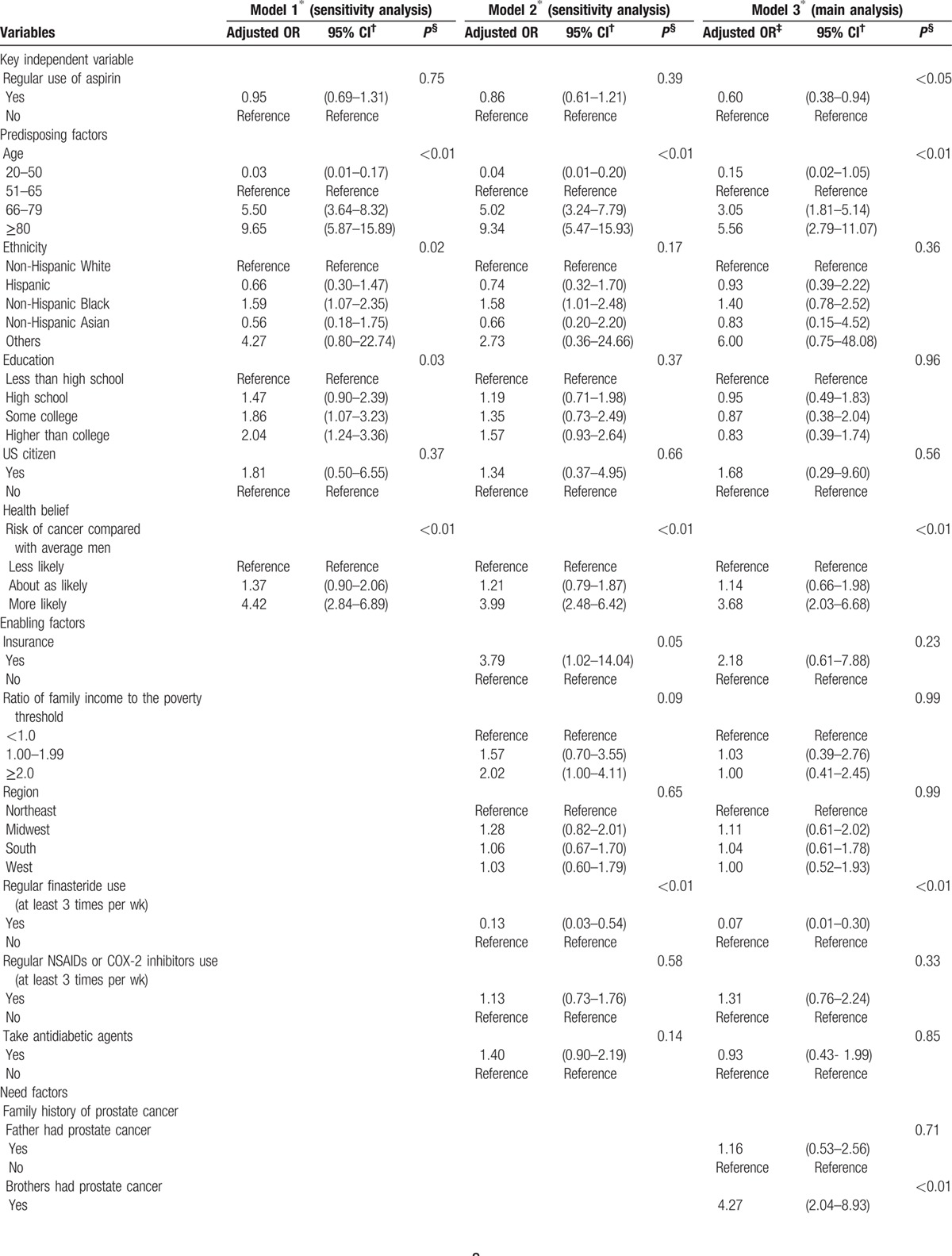
Association between regular use of aspirin and prostate cancer prevalence: results from main and sensitivity analysis by adding predisposing, enabling, and need factors in a hierarchical pattern.

**Table 3 (Continued):**
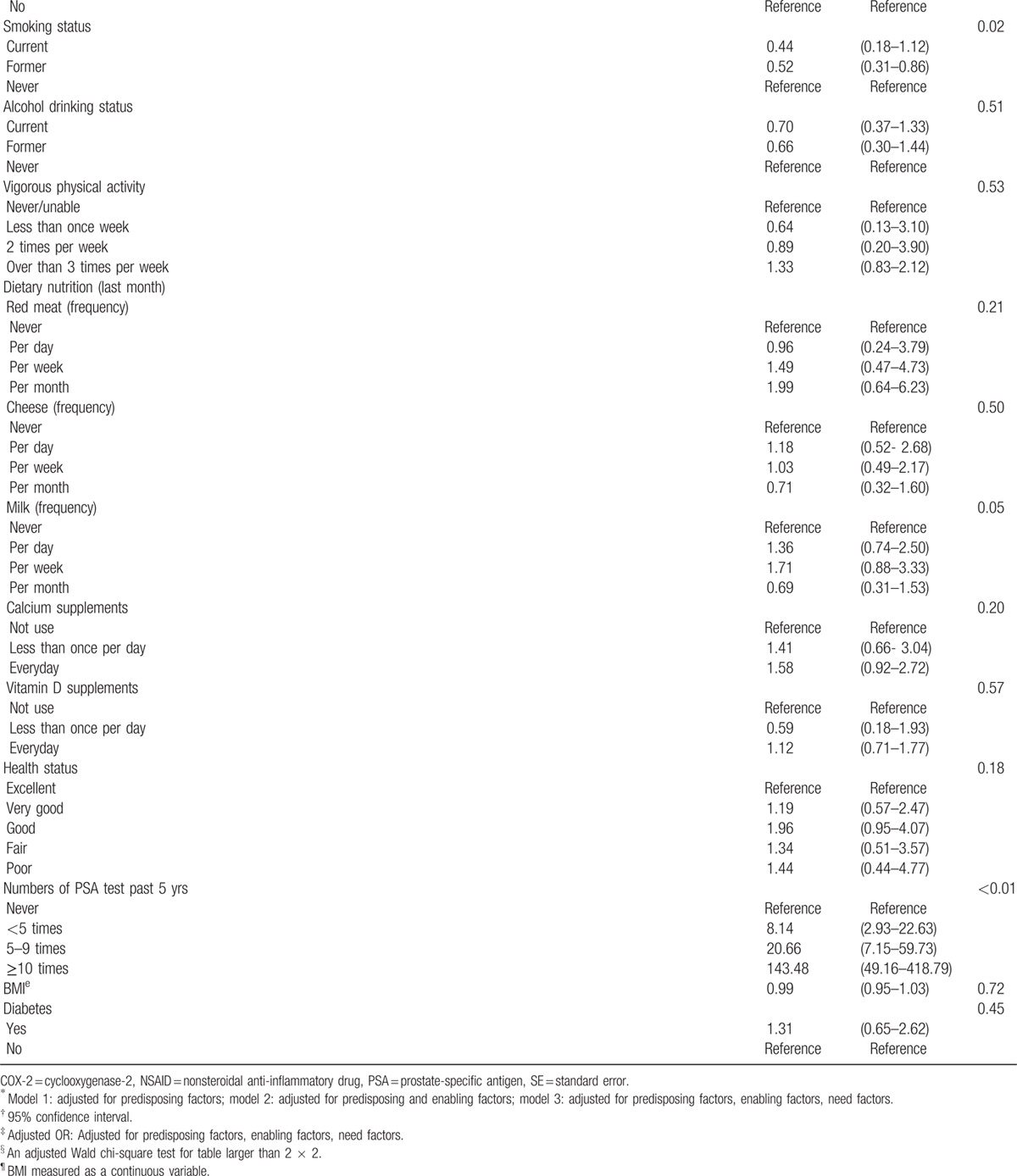
Association between regular use of aspirin and prostate cancer prevalence: results from main and sensitivity analysis by adding predisposing, enabling, and need factors in a hierarchical pattern.

## 4 Discussion

To the best of our knowledge, this is the first study to evaluate the association between regular aspirin use and the prevalence of prostate cancer in a national sample of the US male population. This is also the first study to use Andersen Behavioral Model of Health Services Use to comprehensively select risk factors and assess their associations with the risk of prostate cancer. In our study, regular aspirin use was found to be significantly associated with a lower prevalence of prostate cancer when compared with nonregular aspirin use among the US male population in 2010.

Previous studies have reported a similar finding that aspirin is associated with a reduced risk of prostate cancer.^[16,18]^ In contrast to past studies, 1 advantage of our study is that it comprehensively included several covariates to fully adjust the effect of regular aspirin use on the risk of prostate cancer. These covariates included are as follows: (1) nutritional variables, such as red meat, milk, cheese, calcium, and vitamin D supplements; (2) lifestyle factors, such as exercise, smoking habits, and alcohol consumption; and (3) health belief of cancer risk. These covariates, which were not fully adjusted in past studies,^[16–18]^ were assumed to be risk factors to be associated with prostate cancer. Furthermore, our study is a theory-based study in which Andersen Behavioral Model of Health Services Use was used as a theoretical framework to guide the process of covariate selection.

In our study, we did not find a significant association between NSAID use and the reduced prevalence of prostate cancer. Whether the use of NSAIDs could reduce the risk of prostate cancer remains unclear, and findings from previous studies were controversial.^[16,17,62–64]^ For example, Jacobs et al^[17]^ used the American Cancer Society's Cancer prevention Study II Nutrition cohort to evaluate the impact of aspirin and NSAID use on prostate cancer incidence. They found that regular use of NSAIDs in the past 5 or more years was associated with a reduced risk of prostate cancer, but current NSAID use was not associated with decreased prostate cancer risk.^[17]^ The measurement of NSAID use in our study was based on self-report reflecting whether respondents reported current use of nonaspirin NSAIDs or COX-2 inhibitors. Similar to the Jacobs et al's study,^[17]^ we did not find a significant association between NSAID use and a reduced risk of prostate cancer. This might be attributed to the specificity of the survey questions. The question was originally phrased as “Do you now take any of the following medications regularly, that is, at least 3 times a week… Advil, Ibuprofen, Motrin, Nuprin, Aleve, Naprosyn, Naproxen, or Celebrex?” The question mixed traditional NSAIDs and more selective NSAIDs such as Celebrex together and it was not possible to distinguish the effect of each NSAID on the risk of prostate cancer. Therefore, NSAIDs, unlike aspirin, could have differing mechanisms, which may or may not be associated with a lower prevalence of prostate cancer. Therefore, further research is necessary to determine whether the use of NSAIDs is associated with reduction in the prevalence of prostate cancer.

Several covariates such as patients’ health belief, family history, age, smoking status, regular finasteride use, and the number of PSA tests in the past 5 years were also found to be associated with a lower prevalence of prostate cancer. For example, respondents with a belief that they were more likely to get cancer had a higher self-reported prevalence of prostate cancer. Patients with a strong cancer health belief could be more likely to have a prostate screen, which can result in a higher likelihood of being diagnosed with prostate cancer.^[65]^ Regarding family history, we found respondents whose brothers had prostate cancer were associated with a higher self-reported prevalence of prostate cancer. Similar to previous studies, family history is a risk factor for prostate cancer.^[20,40,48]^

Moreover, we found respondents who had a higher number of PSA tests in the past 5 years had an increased prevalence of prostate cancer. Because PSA testing has increased in the past decade,^[21,55]^ the incidence of prostate cancer is rising proportionately through earlier diagnosis. This might be countered by the argument that there is high detection bias with the PSA test,^[21,54,55]^ but respondents who received PSA testing more frequently still had a higher likelihood of being diagnosed with prostate cancer.

Diet has been considered as a possible risk factor for prostate cancer.^[19,36,51]^ In our study, we did not find dietary factors to be associated with an increased prevalence of prostate cancer. The discrenpacy between our findings and previous findings could be a function of the measurment of the dietary covariates, and therefore such a relationship cannot be ruled out completely.

Finally, we found finasteride use was associated with a lower prevalence of prostate cancer. In a 7-year trial, finastate, a 5 alpha-reductase inhibitor that was used to treat benign prostatic hyperplasia (BPH) and male pattern baldness (MPB), was found to be associated with a lower risk of prostate cancer by inhibition of the conversion of testosterone to the more potent androgen dihydrotestosterone within the prostate.^[23]^ Respondents who self-reported regular use of finasteride had a lower prevalence of prostate cancer in our study.

A number of limitations should be noted when interpreting these results. Similar to all survey data, recall bias could not be eliminated in our study. Moreover, this is a cross-sectional study. The temporal ambiguity and protopathic bias, which refers to bias from reverse causation, can still exist in our study. Due to the cross-sectional nature of our study, only the association, but not the causality, between the variables can be drawn from the findings. The prevalence of prostate cancer in our study was based on respondent self-report. Respondents reported whether they were told by a doctor or a health professional that they were diagnosed with prostate cancer. However, no confirmation of this diagnosis was made. The diagnosis made by only 1 health professional without external confirmation can raise potential inaccuracy in the diagnosis. Although NHIS is a valid and reliable survey,^[66,67]^ the inaccuracy of diagnosis due to the questionnaire design could still threaten the validity of our study. Further, for some questions, we cannot determine the rationale for respondents’ self-reported answers. For example, we were not able to determine if a respondent having multiple PSA tests in the past 5 years was for preventive purposes or a consequence of prostate cancer. Because we used existing survey data, we did not have ideal information on medications, such as statins,^[68,69]^ metformin,^[45,46,70]^ or dutasteride,^[71]^ which may potentially be associated with a reduced risk of prostate cancer. Unmeasured confounding related to these medications may exist. However, we adjusted for finasteride use, which has a similar anticarcinogenic effect in prostate cancer cells as dutasteride.^[22,23]^ Thus, the confounding effect from dutasteride would be minimal. Finally, we could not obtain medication use information regarding dosage and duration of aspirin use.

Our study provides a first step to evaluate the association between regular aspirin use and a lower self-reported prevalence of prostate cancer in the United States. A longitudinal study with a longer follow-up period, and also detailed dosage and intake duration information, is necessary. To further investigate the association between regular aspirin use and prostate cancer, future research could be conducted by using a randomized controlled study design, or more efficiently by using longitudinal administrative claims data.

## 5 Conclusions

Our study was based on a nationally representative sample of rich survey data. The results indicated that regular aspirin use was found to be significantly associated with a lower self-reported prevalence of prostate cancer in the US male population in 2010. Further clinical trials and cohort studies with a longer study period are merited to investigate the mechanism and confirm the causality between regular aspirin use and prostate cancer risk.
